# Supplementary material for: Decreased Expression of Estrogen Receptors Is Associated with Tumorigenesis in Papillary Thyroid Carcinoma
Source: Int J Mol Sci. 2022 Jan 18;23(3):1015. doi: 10.3390/ijms23031015 (PMC8835567; doi:10.3390/ijms23031015)
Supplement: Supplementary file 1 [file ijms-23-01015-s001.zip › ijms-1508593-supplementary.pdf]

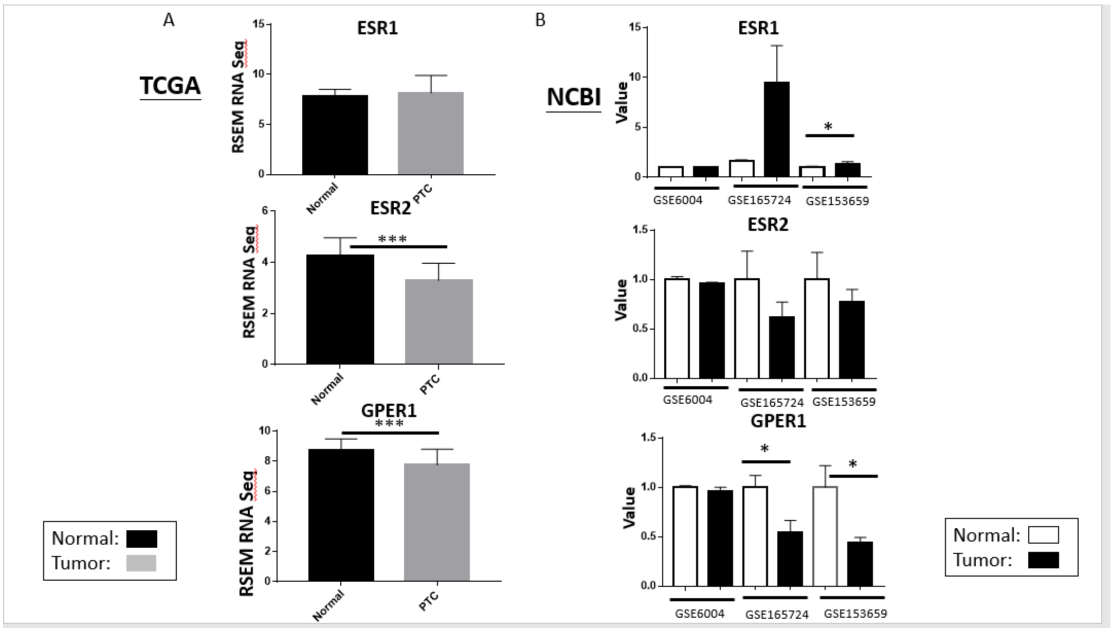


**Figure S1.** Low ERβ and *GPER1* mRNA expression levels in PTC specimens from open access datasets. Analysis of ERα, ERβ, and *GPER1* mRNA expression levels in (**A**) TCGA datasets and (**B**) NCBI datasets. ER mRNA expression levels was presented as RNA-seq RSEM (log2(norm count + 1)) in TCGA datasets. *** $p < 0.001$ * $p < 0.05$ compared with normal thyroid tissues.

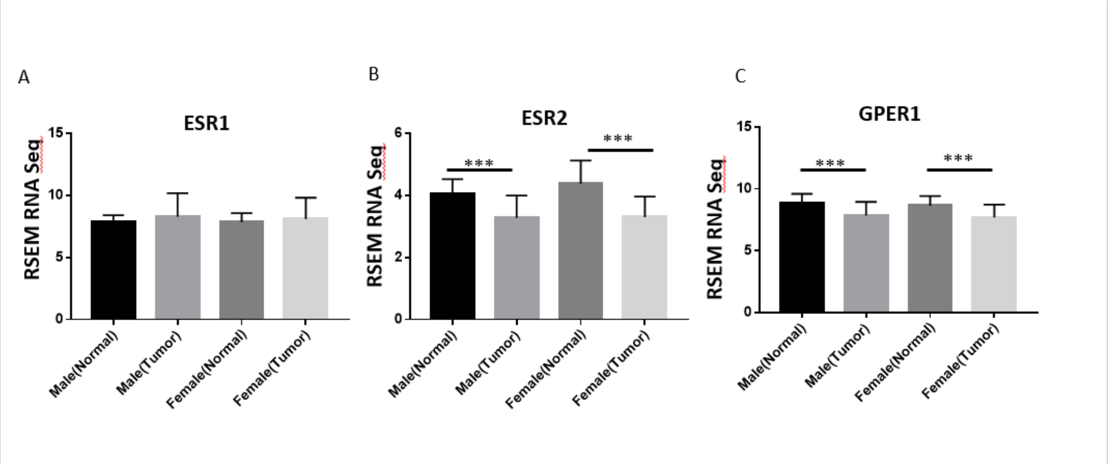


**Figure S2.** Low mRNA expression levels of ERβ and *GPER1* in PTC specimens from both male and female patients. Expression levels of (**A**) ERα, (**B**) ERβ, and (**C**) *GPER1*. The mRNA expression levels of ERs was labeled as RNA-seq RSEM (log2(norm count + 1)) in TCGA datasets. * $p < 0.00$ compared with normal thyroid tissues.*** $p < 0.001$ compared with normal thyroid tissues.
